# Chemical tuning of dynamic cation off-centering in the cubic phases of hybrid tin and lead halide perovskites†
†Electronic supplementary information (ESI) available: Details of the sample synthesis. LeBail fits of the X-ray diffraction data at 360 K. Fourier transform optimization of the X-ray total scattering data. Cubic fits of the XPDF data over 10 Å to 20 Å. Fits of the XPDF data over 2 Å to 5 Å against all models. Cubic and rhombohedral fits of APbBr_3_ at 300 K and 360 K. See DOI: 10.1039/c7sc01429e



**DOI:** 10.1039/c7sc01429e

**Published:** 2017-06-16

**Authors:** Geneva Laurita, Douglas H. Fabini, Constantinos C. Stoumpos, Mercouri G. Kanatzidis, Ram Seshadri

**Affiliations:** a Materials Research Laboratory , University of California , Santa Barbara , California 93106 , USA; b Materials Department , University of California , Santa Barbara , California 93106 , USA; c Department of Chemistry and Biochemistry , University of California , Santa Barbara , California 93106 , USA . Email: seshadri@mrl.ucsb.edu; d Department of Chemistry , Argonne-Northwestern Solar Energy Research (ANSER) Center , Northwestern University , Evanston , Illinois 60208 , USA

## Abstract

Hybrid halide perovskites combine ease of preparation and relatively abundant constituent elements with fascinating photophysical properties. Descriptions of the chemical and structural drivers of the remarkable properties have often focused on the potential role of the dynamic order/disorder of the molecular A-site cations. We reveal here a key aspect of the inorganic framework that potentially impacts the electronic, thermal, and dielectric properties. The temperature evolution of the X-ray pair distribution functions of hybrid perovskites ABX_3_ [A^+^ = CH_3_NH_3_ (MA) or CH(NH_2_)_2_ (FA); B^2+^ = Sn or Pb; X^–^ = Br, or I] in their cubic phases above 300 K reveals temperature-activated displacement (off-centering) of the divalent group 14 cations from their nominal, centered sites. This symmetry-lowering distortion phenomenon, previously dubbed *emphanisis* in the context of compounds such as PbTe, is attributed to Sn^2+^ and Pb^2+^ lone pair stereochemistry. Of the materials studied here, the largest displacements from the center of the octahedral sites are found in tin iodides, a more moderate effect is found in lead bromides, and the weakest effect is seen in lead iodides. The A-site cation appears to play a role as well, with the larger FA resulting in greater off-centering for both Sn^2+^ and Pb^2+^. Dynamic off-centering, which is concealed within the framework of traditional Bragg crystallography, is proposed to play a key role in the remarkable defect-tolerant nature of transport in these semiconductors *via* its effect on the polarizability of the lattice. The results suggest a novel chemical design principle for future materials discovery.

## Abstract 2

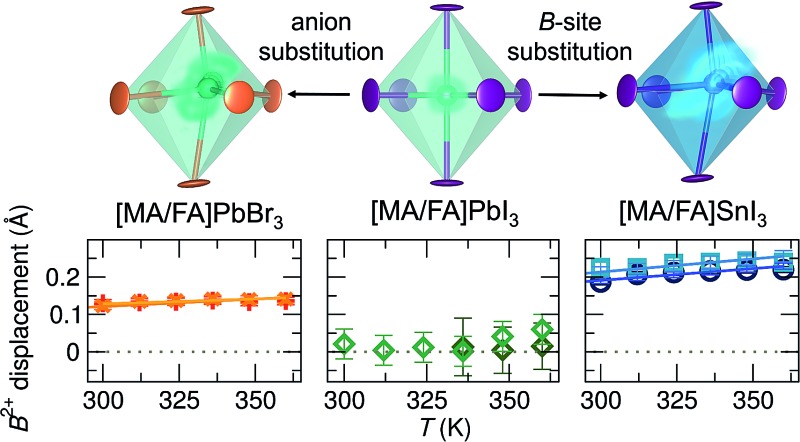
We reveal here a key aspect of the inorganic framework of hybrid halide perovskites that potentially impacts the electronic, thermal, and dielectric properties.

## 1 Introduction

Inorganic and hybrid organic–inorganic halide perovskites ABX_3_ [A^+^ = Cs, CH_3_NH_3_ (MA), or CH(NH_2_)_2_ (FA); B^2+^ = Ge, Sn, or Pb; X^–^ = Cl, Br, or I] have attracted significant research attention of late due to their impressive optoelectronic performance, ease of preparation, and abundant constituent elements. Since the first application of hybrid lead iodides in photovoltaic (PV) devices in 2009,^1^ the conversion efficiency of record perovskite PV cells has risen to over 20%,^2^ and the field has broadened substantially to include the pursuit of lead-free materials,^3–11^ bromides and mixed halides for light emission and detection applications,^12–17^ and layered perovskite-derivatives for enhanced stability.^18–20^ However, key aspects regarding the origins of the remarkable functionality of these materials remain enigmatic. Among them are: why do the low rates of carrier trapping and recombination^16,17,21–23^ in these relatively soft, solution-processed materials approach those of the best high purity III–V semiconductors, leading to long carrier lifetimes and long diffusion lengths?^21,24^ Why are the carrier mobilities so modest^21,22,25^ relative to the calculated carrier effective masses and typical scattering rates? In answering these questions, much attention has focused on the potential importance of the A-site organic molecular cations in the hybrid systems,^26–28^ but recent reports suggest that all-inorganic analogues exhibit many of the same structural tendencies^29,30^ and favorable transport properties.^23,31–34^

A clue then to the unusual properties potentially lies in these systems being proximate in phase space (composition, temperature, pressure, strain) to symmetry-lowering distortions of the octahedral coordination environment of the group 14 divalent cation. The presence of this instability and the role that it may play in the properties of the perovskites have been hinted at in prior reports,^29,35–37^ but have not been explored in detail, nor have the impacts on the properties been fully considered. The strength of this effect is dictated by the stability of the ns^2^ level of the isolated lone-pair bearing cation and the electronegativity of the halogen. In the case of the perovskites studied here, the Goldschmidt tolerance factor, as influenced by the size of the A-site cation and potentially the shape of the A cation, is also likely to play a role, providing guidelines for tuning these effects in a rational manner.

Main-group cations with a valence state that is two fewer than the group valence (*e.g.* Sn^2+^, Sb^3+^, Tl^+^, Pb^2+^ and Bi^3+^) possess the lone pair s^2^p^0^ electronic configuration and are prone to symmetry-lowering distortions associated with the pseudo- or second-order-Jahn–Teller effects.^38,39^ Heavier cations have deep ns^2^ levels due to relativistic stabilization, reducing the strength of the on-site s–p hybridization. The higher energy of the ns^2^ levels of lighter cations, as well as the mixing with the anion p orbitals, leads to stereochemical expression of the lone pair.^40–44^ This is exemplified in the AGeI_3_ (A^+^ = Cs, MA, FA) perovskite analogs,^45^ where the strong tendency for the activity of the 4s^2^ electrons of Ge results in room-temperature structures with highly distorted Ge environments, which also happen to be polar. In contrast, the heavier Sn and Pb atoms form halide perovskite compounds that can crystallize with these cations in nominally regular octahedral environments.

The choice of ligand is also a key factor with more electronegative anions resulting in greater interaction of the anion p states with the orbitals of the cation and thus greater propensity for the off-centered coordination polyhedra associated with lone pair stereochemical activity.^43^ When the propensity for lone pair stereochemical activity is not sufficiently strong relative to the thermal energy to produce a ferroically distorted phase, uncorrelated, local off-centering displacements of the main-group cation can result. Such displacements have recently been observed to emerge from a high symmetry phase upon heating in rock-salt group 14 chalcogenides,^46–48^ and this phenomenon has been termed *emphanisis*. This phenomenon leads to substantial anharmonicity of the lattice dynamics, contributing to the observed ultralow thermal conductivity important for thermoelectrics,^49^ and has been a topic of intense interest since the first reports.^50–57^ Our recent work has shown a similar dynamic displacement of Sn^2+^ in CsSnBr_3_ at ambient and elevated temperatures,^29^ suggesting that an emphanitic local distortion of the metal halide network may also be present in the technologically important hybrid halide perovskites.

We show here that the tendency for symmetry-lowering local distortions of the group 14 cation coordination environment exists across the hybrid halide perovskites. The pair distribution functions for ABX_3_ (A^+^ = MA or FA; B^2+^ = Sn or Pb; X^–^ = Br or I) calculated from X-ray scattering at and above 300 K reveal temperature-activated, dynamic off-centering of the lone pair-bearing Sn^2+^ and Pb^2+^ cations that is not observed through traditional crystallographic techniques. This local off-centering is described by displacements along <111>, and we find the largest degree of off-centering in lead-free tin iodides, a moderate degree in lead bromides, and the smallest degree in the lead iodide compositions. This qualitative agreement with the chemical predictors of the lone pair stereochemical activity as enumerated for the group 14 chalcogenides,^43^ together with our prior *ab initio* studies of the perovskite CsSnBr_3_,^29^ implicates the lone pairs as the driving force for this behavior. This phenomenon has profound implications on understanding the properties: systems displaying this proximal instability exhibit strongly anharmonic lattice dynamics leading to an elevated static dielectric response, which reduces carrier scattering, trapping, and recombination,^58–60^ as well as high coefficients of volumetric thermal expansion^29,61–63^ and unusual temperature evolution of the bandgap.^29^ The substantial lattice polarizability associated with this proximal instability is of particular importance as it may explain why the carrier mobilities are limited by scattering from phonons rather than charged defects^22,64,65^ and why carrier trapping and recombination rates are so low.^21–23^ This additionally lends credence to the hypothesis of large polaron formation^60^ that reconciles the small carrier effective masses from band theory with the modest mobilities observed in the experiment. Chemical control of this phenomenon, as demonstrated by the qualitative composition trends observed here, offers new design principles in the search for defect-tolerant semiconductors.

## 2 Experimental section

### 2.1 Sample synthesis

The hybrid perovskites were prepared following modifications of the previously reported procedures.^66^ PbO and CH_3_NH_3_Cl were purchased from Sigma-Aldrich. HC(NH_2_)_2_Cl was prepared by stoichiometric addition of solid HC(NH_2_)_2_(O_2_CCH_3_) in 37% aqueous HCl, followed by rotary evaporation and washing with toluene to remove excess acetic acid.^63^ Black SnO was prepared following a modification of the literature procedure.^67^ The detailed procedures for the preparation of the polycrystalline samples of CH_3_NH_3_PbI_3_, HC(NH_2_)_2_PbI_3_, CH_3_NH_3_SnI_3_, HC(NH_2_)_2_SnI_3_, CH_3_NH_3_PbBr_3_, and HC(NH_2_)_2_PbBr_3_ can be found in the ESI.†

### 2.2 X-ray scattering data collection and modeling

For the synchrotron total scattering measurements, samples of fine powder, obtained by the means described above, were transferred into Kapton capillaries (0.81 mm OD, 0.8 mm ID) and tightly compacted to ensure a maximum packing fraction. Both ends of the capillaries were sealed with epoxy and stored in a N_2_ atmosphere prior to the measurement.

The synchrotron X-ray total scattering measurements were recorded on the 11-ID-B beam line at the Advanced Photon Source located at Argonne National Laboratory. The 2D scattering data were collected on a Perkin-Elmer amorphous Si-based area detector. A photon wavelength of 0.2114 Å (58.66 keV) was used for MAPbI_3_ (collected from 360 K to 300 K), FAPbI_3_ (collected from 480 K to 300 K to ensure conversion from the yellow *δ* phase to the black perovskite phase, and verified by analysis of the reciprocal space data, shown in Fig. S1† at 360 K), MAPbBr_3_ (collected from 360 K to 300 K), and FAPbBr_3_ (collected from 360 K to 300 K). A photon wavelength of 0.1430 Å (86.70 keV) was used for MASnI_3_ (collected from 360 K to 300 K) and FASnI_3_ (collected from 360 K to 300 K) to avoid the Sn fluorescence edge at 29.21 keV. The data were collected every 2 minutes upon cooling at a rate of 6 K min^–1^. Fit2D^68^ was utilized to integrate the 2D data to the 1D diffraction patterns. Corrections to obtain the *S*(*Q*) and subsequent Fourier transform with a *Q*_max_ of 23 Å^–1^ and an *r*-grid of 0.01 Å to obtain the X-ray pair distribution function (PDF, *G*(*r*)) were performed using the program PDFgetX2.^69^ These parameters were chosen to optimize the *r*-resolution while minimizing the Fourier termination ripples satisfactorily for all samples across the series, and an example of the optimization process is shown in Fig. S2† for FASnI_3_. The instrumental parameters used in the fits were *Q*_broad_ = 0.06 Å^–1^ and *Q*_damp_ = 0.01 Å^–1^, as determined from a CeO_2_ standard.

For all samples, the A-site cations were modeled as a pseudo-atom with an equivalent scattering power (K for CH_3_NH_3_ and Mn for CH(NH_2_)_2_) placed in the center of the A site at (0, 0, 0) and given a large (between 0.2 and 0.4 Å^2^) atomic displacement parameter (ADP). For all fit ranges, the fits of the XPDF data were first performed against the cubic *Pm*3*m* model to obtain the lattice parameters. The ADPs for the A- and B-site cations were refined isotropically, while the ADPs for the halide atoms were allowed to refine anisotropically. The cubic space groups were subsequently transformed into the respective *I*4*cm* and *R*3*m* space groups using the “TRANSTRU” tool on the Bilbao Crystallographic Server.^70–72^ Fits against the *I*4*cm* model were first performed over an *r*-range of 2 Å to 25 Å. The lattice parameters were fixed to the transformed values from the cubic fit. The halide positions and anisotropic ADPs were allowed to refine, while the A-site ADPs were fixed to those obtained from the cubic fit. The B-site ADPs were fixed to 0.008 Å^2^. For fits against the remaining *r*-ranges (2 Å to 5 Å and all incremental fits), the lattice parameters were fixed to the values obtained from the transformed structures, the halide ADPs and displacements were fixed to the values obtained from the 2 Å to 25 Å fits while the B-site displacement in the 4a Wyckoff position (0, 0, *z*) was allowed to refine. Fits against the *R*3*m* model were first performed over an *r*-range of 2 Å to 25 Å. The lattice parameters were fixed to the transformed values from the cubic fit. The halide anisotropic ADPs were allowed to refine, while the A-site ADPs were fixed to those obtained from the cubic fit. The B-site ADPs were fixed to 0.008 Å^2^. For fits against the remaining *r*-ranges (2 Å to 5 Å and all incremental fits), the lattice parameters were fixed to the values obtained from the transformed structures, the halide ADPs were fixed to the values obtained from the 2 Å to 25 Å fits while the B-site displacement in the 3a Wyckoff position (0, 0, *z*) was allowed to refine.

## 3 Results & discussion

**Fig. 1:**
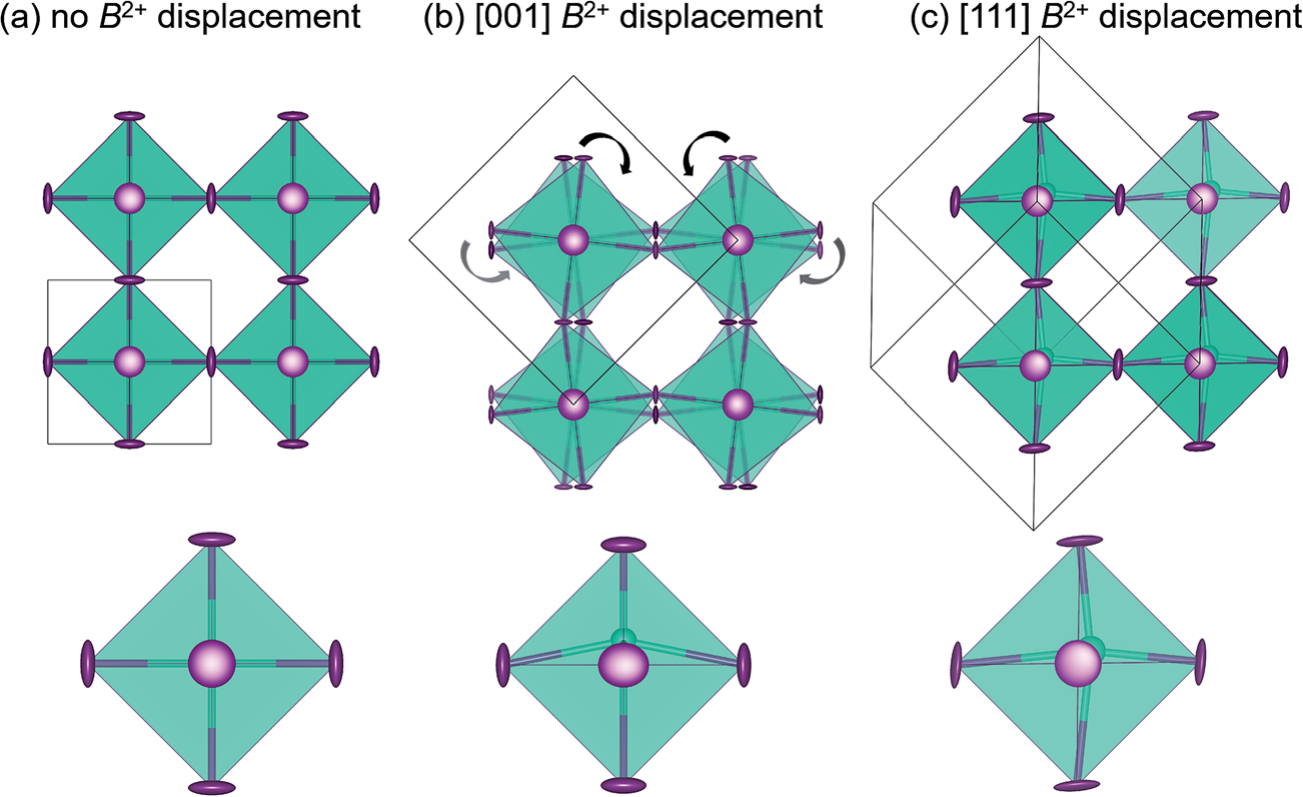
Crystal structures chosen to model the X-ray PDF data: (a) cubic *Pm*3*m* with no B-site off-centering or octahedral rotations, (b) tetragonal *I*4*cm* with allowed B-site off-centering and static octahedral rotations, and (c) rhombohedral *R*3*m* with allowed B-site off-centering and no octahedral rotations.

Dynamic octahedral rotations have been observed through local techniques at elevated temperatures in several halide perovskite systems^29,37^ which may be active in conjunction with dynamic off-centering of the B-site cations. To investigate the interplay between the octahedral rotations and B-site stereochemical activity (and subsequent off-centering), three crystallographic models (Fig. 1) were chosen to fit against the X-ray PDF data over the various *r*-ranges: cubic *Pm*3*m*, which allows neither octahedral rotations nor B-site off-centering; tetragonal *I*4*cm*, which allows for simultaneous octahedral rotations and B-site off-centering along the *c*-axis; rhombohedral *R*3*m*, which allows for no octahedral rotations but does allow for B-site off-centering along the [111] crystallographic direction. The space groups chosen to model the local symmetry of the PDF data were based on the crystallographic structures with prototypical ferroic displacements in perovskite systems. In an effort to qualitatively describe the local structure, we have chosen known models that systematically lower the symmetry without drastically increasing the number or correlation of refined parameters. We have previously reported the *R*3*m* structure as an approximate description of the dynamic off-centering of Sn^2+^ in CsSnBr_3_ (in comparison to the *Pm*3*m*, *P*4*mm*, and *Amm*2 models),^29^ while the local coexistence of rotations and Pb^2+^ off-centering described by *I*4*cm* has been reported for crystallographically cubic MAPbI_3_.^37^

**Fig. 2:**
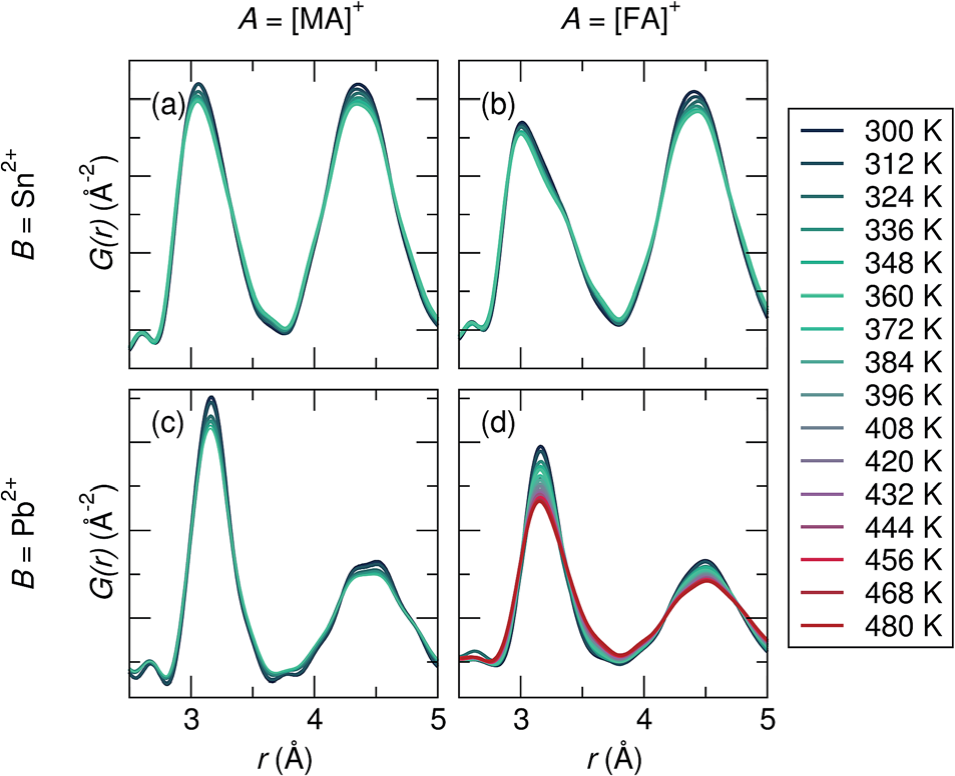
Overlay of the raw X-ray PDF data collected over a range of 300 K to *T*_max_ for (a) MASnI_3_ (*T*_max_ = 360 K), (b) FASnI_3_ (*T*_max_ = 360 K), (c) MAPbI_3_ (*T*_max_ = 360 K), and (d) FAPbI_3_ (*T*_max_ = 480 K). Peak asymmetry of the first B–I correlation at approximately 3 Å is observed in all compositions, but is most pronounced in the Sn compositions.

The X-ray PDF data was analyzed from 300 K to 360 K (480 K for FAPbI_3_), shown in Fig. 2. The temperature at which the cubic perovskite phase is present varies for each composition: for MASnI_3_ *T* > 275 K,^73^ for FASnI_3_ *T* > 250 K,^66^ for MAPbI_3_ *T* > 327 K,^74^ for FAPbI_3_ *T* > 285 K,^63^ for MAPbBr_3_ *T* > 237 K,^74^ and for FAPbBr_3_ *T* > 265 K (unpublished reference). Therefore, quantitative studies were only performed in the known cubic phase regimes of each sample. Qualitatively, for all compositions the first B–I peak becomes broader and more asymmetric upon warming, and the effect is most pronounced for the Sn^2+^ samples. Peak broadening and asymmetry are expected to further increase with higher collection temperatures, which is evidenced in FAPbI_3_, the only composition collected up to 480 K.

Fits against the X-ray PDF for each sample were performed carefully to avoid excessive correlation of the refined parameters, and the modeling is described in detail in the experimental section. To verify that the samples are crystallographically cubic, the X-ray PDF data were fit over an *r*-range of 10 Å to 25 Å against the cubic *Pm*3*m* model at the various reported cubic phase temperatures, and the representative fits for each sample are shown in the ESI Fig. S3.† Reasonable goodness-of-fit (*R*_w_) values (between 8 and 12%) were obtained for all compositions, suggesting that the data are well described by the expected cubic symmetry as we approach the average, crystallographic length scale. The corresponding reciprocal space data from the total scattering experiment, shown in Fig. S1† at 360 K, is additionally consistent with the cubic perovskite structure and does not indicate the presence of any impurity phases.

**Fig. 3:**
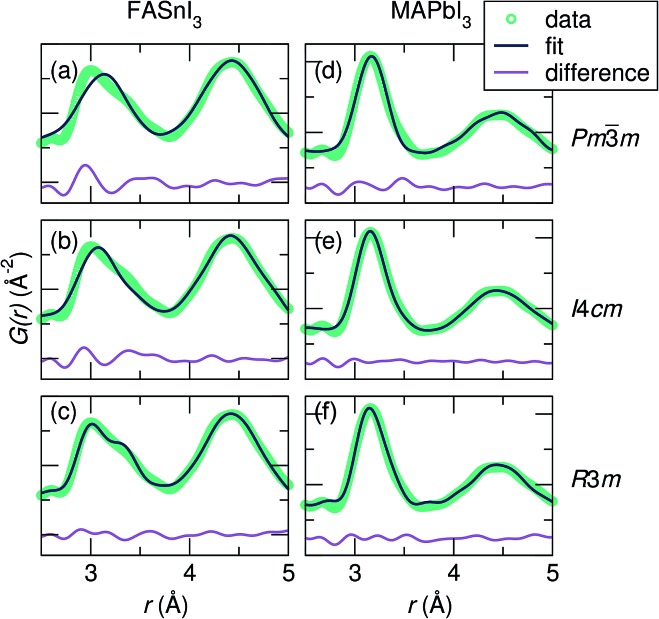
Fits of the X-ray PDF data at 360 K from 2.0 Å to 5.0 Å against the various space groups for representative samples FASnI_3_ [(a) *Pm*3*m*, (b) *I*4*cm*, and (c) *R*3*m*] and MAPbI_3_ [(d) *Pm*3*m*, (e) *I*4*cm*, and (f) *R*3*m*]. For both FASnI_3_ and MASnI_3_ (shown in Fig. S2†), the peak shape of the first Sn–I correlation is best captured by Sn off-centering along <111> as in the *R*3*m* model, while FAPbI_3_ and MAPbI_3_ (shown in Fig. S2†) are equally well-described by both the *I*4*cm* and *R*3*m* models.

Fits of the X-ray PDF data at 360 K against the candidate space group models over a 2.0 Å to 5.0 Å range indicate the poorest fit for all samples against the cubic *Pm*3*m* model. Fits for the most extreme cases, FASnI_3_ (the most distorted B–I peak) and MAPbI_3_ (the least distorted B–I peak), are shown in Fig. 3. For both Sn^2+^ samples, the best description of the Sn–I correlation is with the *R*3*m* model, indicating off-centering best described by rhombohedral symmetry. It should be noted that the goodness-of-fit for the Sn^2+^ compositions is heavily influenced by the fit to the I–I correlations due to the stronger scattering power of I *vs.* Sn (in comparison to I *vs.* Pb). This appears to result in a poorer fit in the Sn–I correlation of these samples; however, this does not change the result that the local symmetry of the Sn^2+^ compositions is best modeled with a rhombohedral off-centering of Sn^2+^. It should also be noted that the use of anisotropic displacement parameters is necessary to fit the I–I correlation around 4.5 Å. However, the implementation of the large anisotropic displacements of the halides perpendicular to the B–I bond does not account for the observed peak asymmetry of the first B–X correlation, even with highly exaggerated anisotropic components, as we demonstrated for CsSnBr_3_.^29^ For both the Pb^2+^ samples, a similar description of the Pb–I correlation is obtained with both the *I*4*cm* and *R*3*m* models. This indicates that cation off-centering is present in the Pb^2+^ samples; however, it is minor compared to that of the Sn^2+^ samples, and complicated by a greater degree of correlation with the refined halide parameters. Regardless, the crystallographic cubic phase is an insufficient model of the local symmetry of the Sn^2+^ and Pb^2+^ coordination environment for all compositions.

**Fig. 4:**
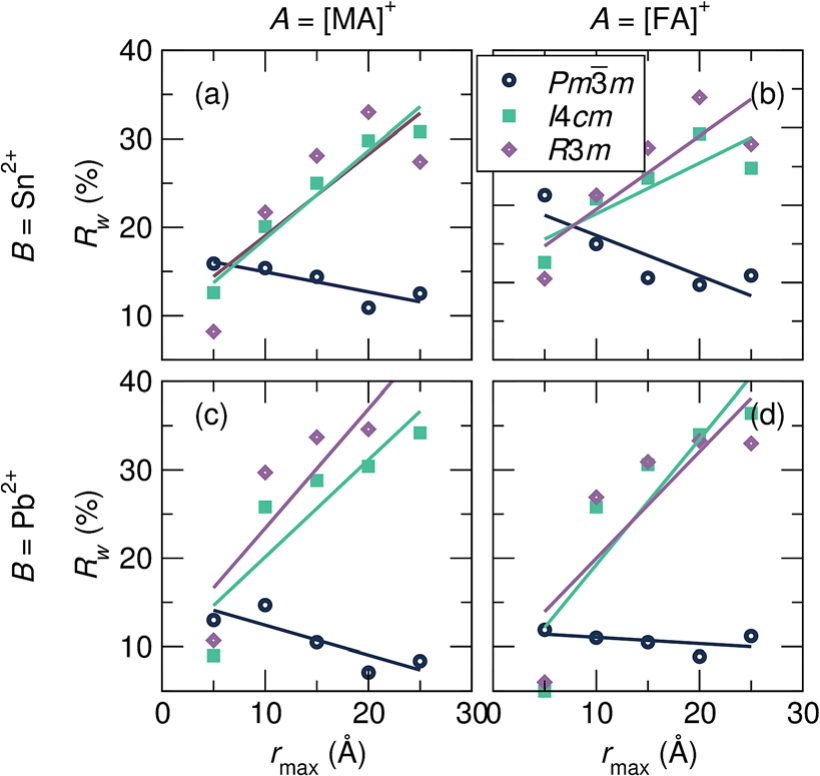
Goodness-of-fit parameters (*R*_w_) as a function of *r*-max for 10 Å incremental fits of the X-ray PDF data at 360 K against the various space group models for (a) MASnI_3_, (b) FASnI_3_, (c) MAPbI_3_, and (d) FAPbI_3_. Models that allow B-site displacements (*I*4*cm* and *R*3*m*) have lower *R*_w_ values with an *r*-max of 5 Å for all samples, while cubic *Pm*3*m* results in lower *R*_w_ values for all fits with an *r*-max of 10 Å and above.

To investigate the coherence length of distortions in the samples, 10 Å incremental fits of the PDF data at 360 K were performed (*r*-ranges = 1 Å to 10 Å, 5 Å to 15 Å, 10 Å to 20 Å, and 15 Å to 25 Å). *R*_w_ values of the fits against the various space group models as a function of *r*_max_ are shown in Fig. 4. *R*_w_ values of the 2 Å to 5 Å fits are also plotted to illustrate the best representation of the local B-site coordination environment. It is observed for all samples that the cubic model quickly becomes the best description of the data as the incremental series progresses, even at an *r*-max of only 10 Å, and the *R*_w_ for the cubic fit continues to decrease with increasing *r*_max_, further illustrating cubic symmetry as the fit range tends towards the average crystallographic structure.

**Fig. 5:**
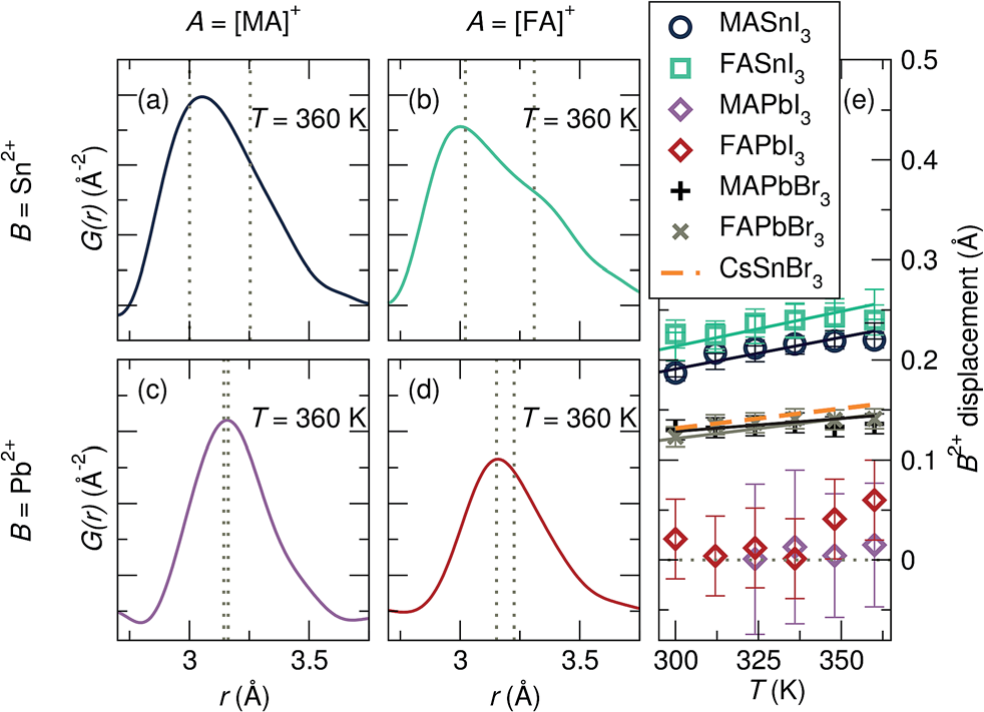
X-ray pair distribution functions for (a) MASnI_3_, (b) FASnI_3_, (c) MAPbI_3_, and (d) FAPbI_3_ at 360 K of the nearest B–I correlation. (e) Refined displacements of the B-site cation in space group *R*3*m* as a function of the temperature indicate large displacements for Sn^2+^ iodides and minimal displacements for Pb^2+^ iodides. Refined displacements for the APbBr_3_ analogs indicate moderate off-centering. Reported Sn^2+^ displacements in CsSnBr_3_ are overlaid for comparison.^29^ The dashed lines in (a–d) indicate the distinct bond lengths extracted from modeling the local structure in the space group *R*3*m* ([111] displacement).

In CsSnBr_3_, we determined the presence of a dynamic displacement of the Sn^2+^ cation of approximately 0.2 Å along the [111] crystallographic direction at 420 K.^29^ Fits of the local structure of the compositions studied herein indicate a similar local cation displacement. The presence of cation displacement with increasing temperature was investigated by fitting the temperature-dependent data of all samples against the *R*3*m* model over a range of 2 Å to 5 Å, shown in Fig. 5. The refined local structures indicate that large displacements are present for the Sn^2+^-containing samples, while they are minimal in the Pb^2+^ samples. At 360 K the maximum displacement for each composition, from largest to smallest, is approximately 0.24 Å in FASnI_3_, 0.22 Å in MASnI_3_, 0.06 Å in FAPbI_3_, and 0.01 Å in MAPbI_3_. This goes with the expected trend of larger displacements in Sn^2+^ than Pb^2+^ due to the larger relativistic effects in Pb and larger displacements with increasing lattice parameter from MA to FA. In addition to these cationic effects, the chemical identity of the anion and subsequent interaction between its orbitals and those of the B-site cation affect the propensity for stereochemical activity. Increasing the hardness of the anion (*i.e.* APbBr_3_ instead of APbI_3_) increases the interaction of the anion p states with the B-site s orbitals,^43^ thus increasing the tendency for activity of the lone pair. Therefore, a larger rhombohedral distortion of Pb^2+^ should be observed in MAPbBr_3_ and FAPbBr_3_ in comparison to their iodide counterparts. Indeed, fitting of the X-ray PDF data of MAPbBr_3_ and FAPbBr_3_ at 360 K against the *R*3*m* model (Fig. S6†) indicates a Pb^2+^ displacement of approximately 0.15 Å. Based on the magnitudes of displacement resulting from the fits, it appears that the identity of the A-site cation, which plays a role in the lattice parameter and influences the octahedral rotations observed upon cooling, has the smallest effect on the stereochemical activation of the lone pair, and the compositions of the B-site cation and X-site anion are the largest drivers for the stereochemical activity of the ns^2^ electrons, highlighting the importance of the B–X cation–anion orbital interaction. However, the displacement magnitudes reported for CsSnBr_3_ (ref. 29) are essentially indistinguishable from those we find for MAPbBr_3_ and FAPbBr_3_, suggesting that the A-site shape may matter in addition to the size, as one would expect greater displacements for Sn^2+^ than for Pb^2+^ given the same anion. We expect these chemical trends to be universal in the halide perovskite materials, even extending to the layered perovskites, which are known to exhibit distortions of the MX_6_ octahedra.^75–78^ However, the dimensionality of layered perovskites may play an additional role in octahedral distortions and warrants further consideration.

## 4 Conclusions

We have shown through the analysis of pair distribution functions calculated from X-ray scattering experiments that dynamic, temperature-activated B-site cation off-centering displacements occur at and above ambient temperature in the hybrid halide perovskites MASnI_3_, FASnI_3_, MAPbI_3_, FAPbI_3_, MAPbBr_3_, and FAPbBr_3_ as a consequence of the lone pair stereochemical activity. The propensity for stereochemical activity can be tuned through chemical substitution on all sites of the ABX_3_ perovskite structure: the substitution of a larger A-site cation (FA^+^ for MA^+^), a lighter B-site cation (Sn^2+^ for Pb^2+^), and a harder anion (Br^–^ for I^–^) all enhance the magnitude of these displacements. This tendency arises directly from the inherent instability of high-symmetry coordination for ns^2^p^0^ cations with significant impact on the properties.^29^

Importantly, these observations are consistent with the emerging hypothesis that the remarkable defect-tolerance of these semiconductors is related to the lattice polarizability, and does not require the strongly dipolar [CH_3_NH_3_]^+^ cation of the hybrid compositions. Recent reports have focused on the possibility of large^60^ and small^79^ polaron formation, and the measurements of lifetimes and recombination constants for both hot^28^ and band-edge^23^ carriers point to important differences and similarities across compositions. Zhu and coworkers find that hot carriers are much longer lived in MAPbBr_3_ and FAPbBr_3_ than in CsPbBr_3_ and ascribe this to the dipolar molecular cations.^28^ However, this ignores the negligible FA dipole moment (0.21 D)^26^ compared to that of MA (2.29 D)^26^ and the fact that, unlike the hybrid compositions, CsPbBr_3_ is tilted (orthorhombic, *a* ^+^ *b* ^–^ *b* ^–^) rather than cubic at room temperature,^31,80^ which will affect the lattice dynamics and polarizability both directly and *via* the suppression of the lone pair stereochemical activity through reduced orbital overlap. In a separate report, Zhu and coworkers find extremely low trapping and recombination constants for the band-edge carriers in all three compositions, suggesting the importance of the lead–halogen sublattice, rather than the molecular cations, for defect tolerance.^23^ Additionally, the large static dielectric response of the halide perovskites is well known^63,81–85^ and likely contributes to the effective screening of charged defects,^86^ as has been postulated for thallium halides^58^ and demonstrated for doped complex oxides.^59^ Our findings are consistent with these ideas, suggesting that the desired optoelectronic properties are in large part a consequence of the behavior of the metal–halogen network.

An analogous system where proximal instabilities impact the transport properties may be seen in SrTiO_3_. This d^0^ system—which has attracted renewed attention in recent years, because of the rich electrical transport phenomena doped variants display, particularly in thin film form^87^—is also subject to off-centering instabilities due to second-order Jahn–teller effects.^39^ However, due to the balance of size effects and the perovskite tolerance factor, the expected off-centering transition is pushed down to low enough temperatures that quantum fluctuations suppress any phase transition to a structure with distorted TiO_6_ octahedra.^88^ The dielectric constant is anomalously high, however, resulting in unusual transport behavior in the doped phases.^89,90^ Most notably, polaronic effects, as in the halide perovskites, have been implicated in yielding a measured effective mass that appears larger than one would expect from band structure calculations.^91^

The elevated polarizability conferred by the proximal instability in Sn^2+^ and Pb^2+^ halide perovskites, together with the shallow nature of the defect states due to the antibonding character of the valence band^92–94^ and the possible separation of the excited carriers in reciprocal space due to the spin–orbit interactions,^95–97^ is proposed to imbue these materials with their remarkable defect-tolerance. Actively profiting from the phenomena of proximal instabilities due to lone pairs offers a new paradigm for the chemical design of defect-tolerant semiconductors: other compounds with lone pair-bearing ions in high symmetry environments may exhibit similarly favorable transport and recombination properties.
