# Supplementary material for: Chemical tuning of dynamic cation off-centering in the cubic phases of hybrid tin and lead halide perovskites
Source: Chem Sci. 2017 Jun 16;8(8):5628–35. doi: 10.1039/c7sc01429e (PMC5621007; doi:10.1039/c7sc01429e)
Supplement: Supplementary file 1 [file SC-008-C7SC01429E-s001.pdf]

Electronic Supplementary Material (ESI) for Chemical Science.
This journal is © The Royal Society of Chemistry 2017

SUPPORTING INFORMATION FOR:

Chemical Tuning of Dynamic Cation Off-Centering in the Cubic Phases of Hybrid Tin and Lead Halide Perovskites

Geneva Laurita,[†] Douglas H. Fabini,[†,‡] Constantinos C. Stoumpos,[¶] Mercouri G. Kanatzidis,[*,¶] and Ram Seshadri[*,†,‡,§]

Materials Research Laboratory

University of California, Santa Barbara, California 93106, USA, Materials Department

University of California, Santa Barbara, California 93106, USA, Department of Chemistry,

and

Argonne-Northwestern Solar Energy Research (ANSER) Center,

Northwestern University, Evanston, Illinois 60208, USA, and Department of Chemistry and Biochemistry

University of California, Santa Barbara, California 93106, USA

E-mail: m-kanatzidis@northwestern.edu; seshadri@mrl.ucsb.edu

*To whom correspondence should be addressed
†Materials Research Laboratory
University of California, Santa Barbara, California 93106, USA
‡Materials Department
University of California, Santa Barbara, California 93106, USA
¶Department of Chemistry, and
Argonne-Northwestern Solar Energy Research (ANSER) Center,
Northwestern University, Evanston, Illinois 60208, USA
§Department of Chemistry and Biochemistry
University of California, Santa Barbara, California 93106, USA

Synthesis Details

$CH_3NH_3PbI_3$: 4.46 g (20 mmol) of PbO were initially dissolved in 15 ml of concentrated aqueous HI (57 % w/w) and the solution temperature was raised and held to boiling (ca. 130 °C) to afford a clear yellow solution. Addition of 1.35 g (20 mmol) of solid CH_3NH_3Cl in the solution resulted in the immediate precipitation of a fine black precipitate. The solution was stirred for 1 min and filtered hot under vacuum. The dry black solid produced 7 g (65 % yield based on Pb) of crystallographically pure material which was used for the scattering experiments.

$HC(NH_2)_2PbI_3$: 4.46 g (20 mmol) of PbO were initially dissolved in 15 ml of concentrated aqueous HI (57 % w/w) and the solution temperature was raised and held to boiling (ca. 130 °C) to afford a clear yellow solution. Addition of 1.61 g (20 mmol) of solid $HC(NH_2)_2Cl$ in the solution resulted in the immediate precipitation of a fine black precipitate. The solution was stirred for 1 min and filtered hot under vacuum. During filtration the black solid turned to yellow completely converting over a period of 5 min to 10 min. The dry yellow solid produced 9 g (71 % yield based on Pb) of crystallographically pure material which was used for the diffraction experiments.

$CH_3NH_3SnI_3$: 2.69 g (20 mmol) of SnO were charged in 15 ml of concentrated aqueous HI (57 % w/w) and 5.1 mL of H_3PO_2 (50 % w/w) were added before the mixture temperature was raised and held to boiling (ca. 130 °C), leading to a clear yellow solution. Addition of 1.35 g (20 mmol) of solid CH_3NH_3Cl in the solution resulted in the immediate precipitation of a fine black precipitate. The solution was stirred for 1 min and filtered hot under vacuum. The dry black solid produced 8 g (75 % yield based on Sn) of crystallographically pure material which was used for the scattering experiments.

$HC(NH_2)_2SnI_3$: 2.69 g (20 mmol) of SnO were charged in 15 ml of concentrated aqueous HI (57 % w/w) and 5.1 mL of H_3PO_2 (50 % w/w) were added before the mixture temperature was raised and held to boiling (ca. 130 °C), leading to a clear yellow solution. Addition of 1.61 g (20 mmol) of solid $HC(NH_2)_2Cl$ in the solution resulted in the immedi-

ate precipitation of a fine black precipitate. The solution was stirred for 1 min and filtered hot under vacuum. The dry black solid produced 9 g (83 % yield based on Sn) of crystallographically pure material which were used for the scattering experiments.

$CH_3NH_3PbBr_3$: 4.46 g (20 mmol) of PbO were initially dissolved in 20 ml of concentrated aqueous HI (48 % w/w) and the solution temperature was raised and held to boiling (ca. 130 °C) to afford a clear yellow solution. Addition of 1.35 g (20 mmol) of solid CH_3NH_3Cl in the solution resulted in the immediate precipitation of a fine orange precipitate. The solution was stirred for 1 min and filtered hot under vacuum. The dry orange solid produced 7.5 g (78 % yield based on Pb) of crystallographically pure material which was used for the scattering experiments.

$HC(NH_2)_2PbBr_3$: 4.46 g (20 mmol) of PbO were initially dissolved in 20 ml of concentrated aqueous HBr (48 % w/w) and the solution temperature was raised and held to boiling (ca. 130 °C) to afford a clear yellow solution. Addition of 1.61 g (20 mmol) of solid $HC(NH_2)_2Cl$ in the solution resulted in the immediate precipitation of a fine orange precipitate. The solution was stirred for 1 min and filtered hot under vacuum. The dry orange solid produced 8.5 g (86 % yield based on Pb) of crystallographically pure material which was used for the scattering experiments.

LeBail fits of the X-ray diffraction data against cubic $Pm\bar{3}m$ at 360 K

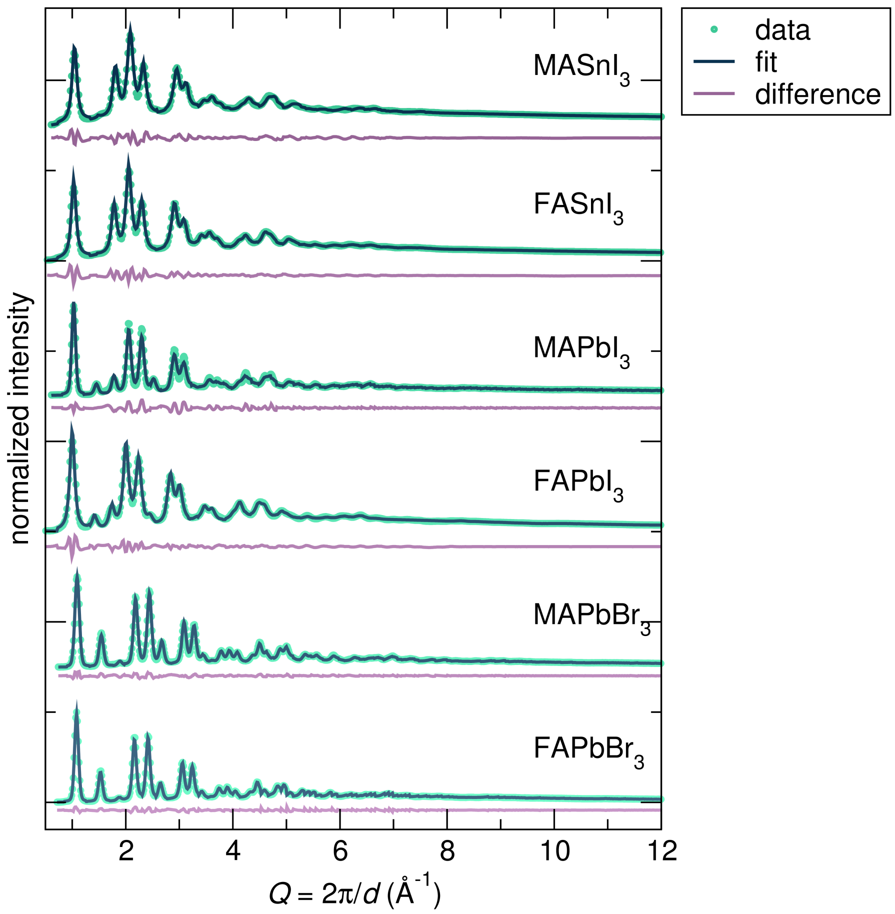

Figure 1: LeBail profile fits of the reciprocal space data at 360 K indicates phase purity. Data for all samples can be indexed to the $Pm\bar{3}m$ space group, indicating all samples are crystallographically cubic at this temperature.

Fourier transform optimization of X-ray total scattering data

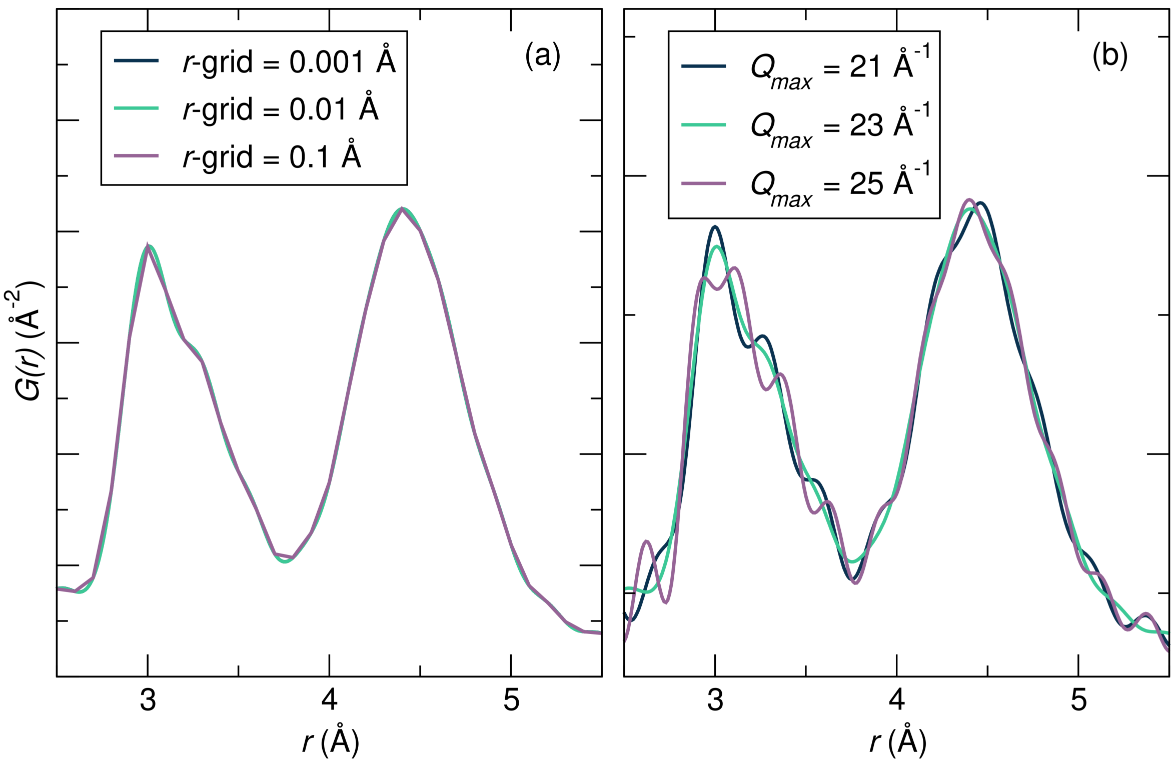

Figure 2: (a) r-grid and (b) Q_{max} series used to determine the optimized Fourier transform parameters of r-grid $= 0.01\,\text{Å}$ and $Q_{max} = 23\,\text{Å}^{-1}$ for representative sample $FASnI_3$ at 360 K.

Cubic fits of the XPDF data over 10 Å to 20 Å

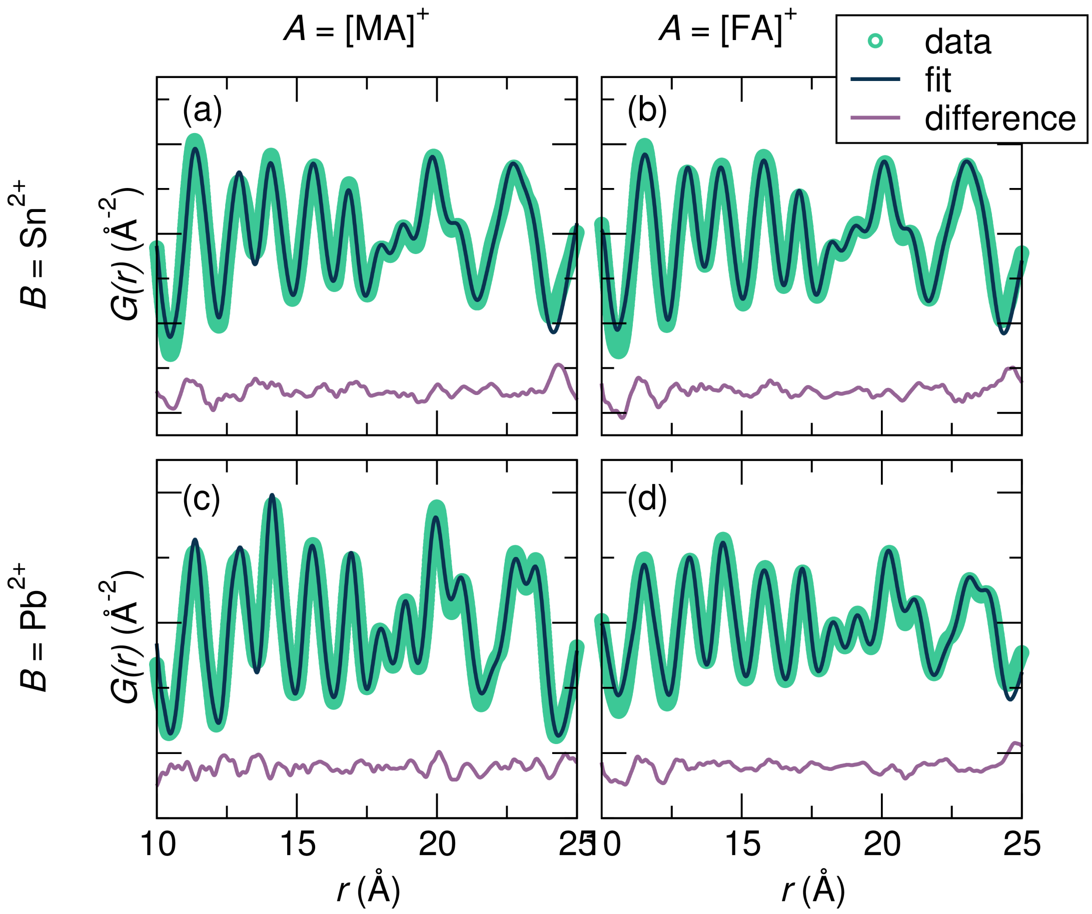

Figure 3: Fits of the synchrotron X-ray PDF data over an r-range of 10 Å to 20 Å against the cubic model with space group $Pm\overline{3}m$ for (a) $MASnI_3$, (b) $FASnI_3$, (c) $MAPbI_3$, and (d) $FAPbI_3$. Fits indicate the data is reasonably described by the cubic model as fit range approaches a crystallographic length scale.

Fits of the XPDF data over 2 Å to 5 Å against all models

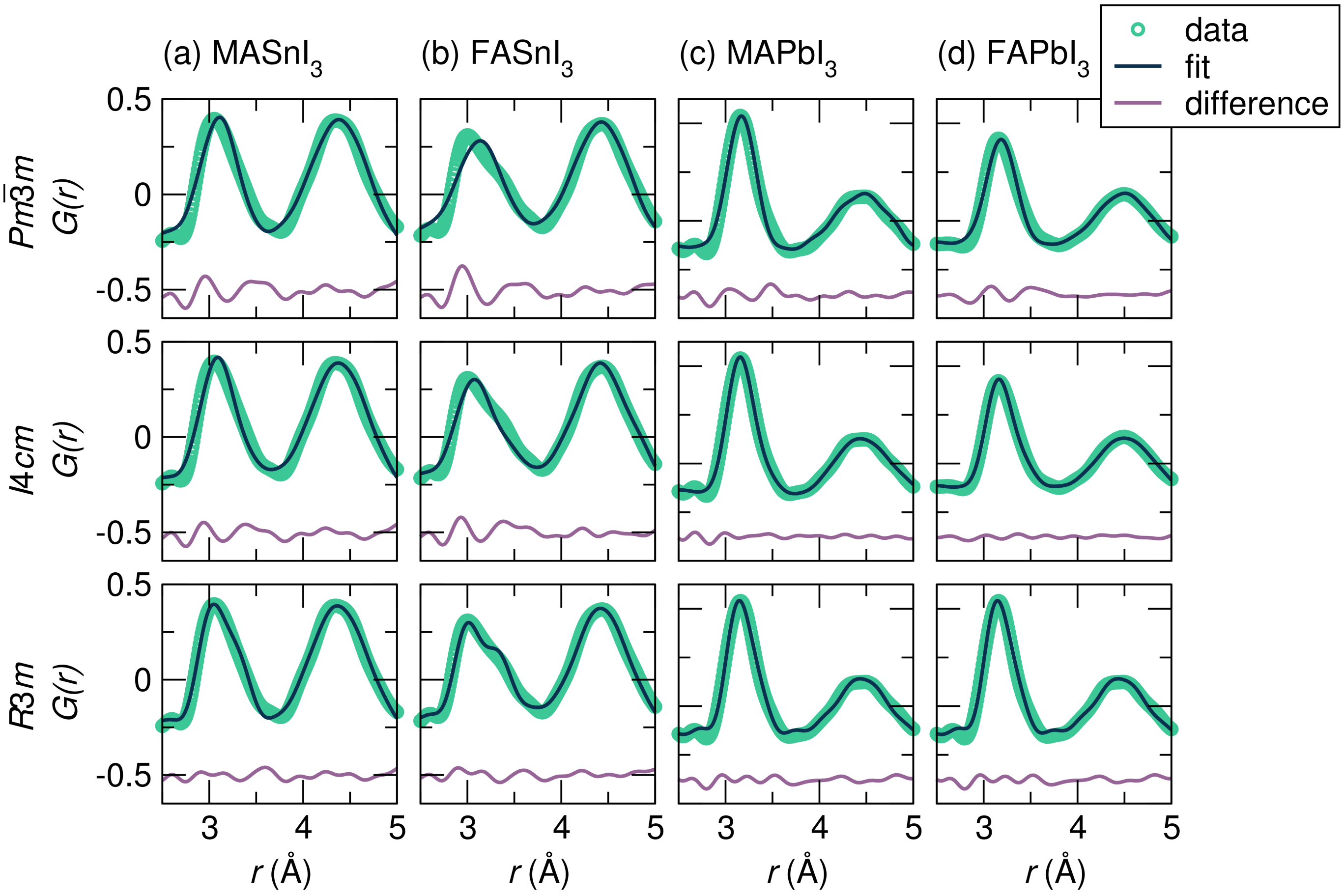

Figure 4: Fits of the synchrotron X-ray PDF data over an r-range of 2 Å to 5 Å against the various space group models $Pm\overline{3}m$ (top row), $I4cm$ (middle row), and $R3m$ (bottom row) for (a) $MASnI_3$, (b) $FASnI_3$, (c) $MAPbI_3$, and (d) $FAPbI_3$. For both Sn^{2+} samples, the local structure is best described by the $R3m$ model, while similar fits are obtained in the Pb^{2+} samples with both the $I4cm$ and $R3m$ models.

Cubic fits of APbBr$_3$ at 300 K and 360 K

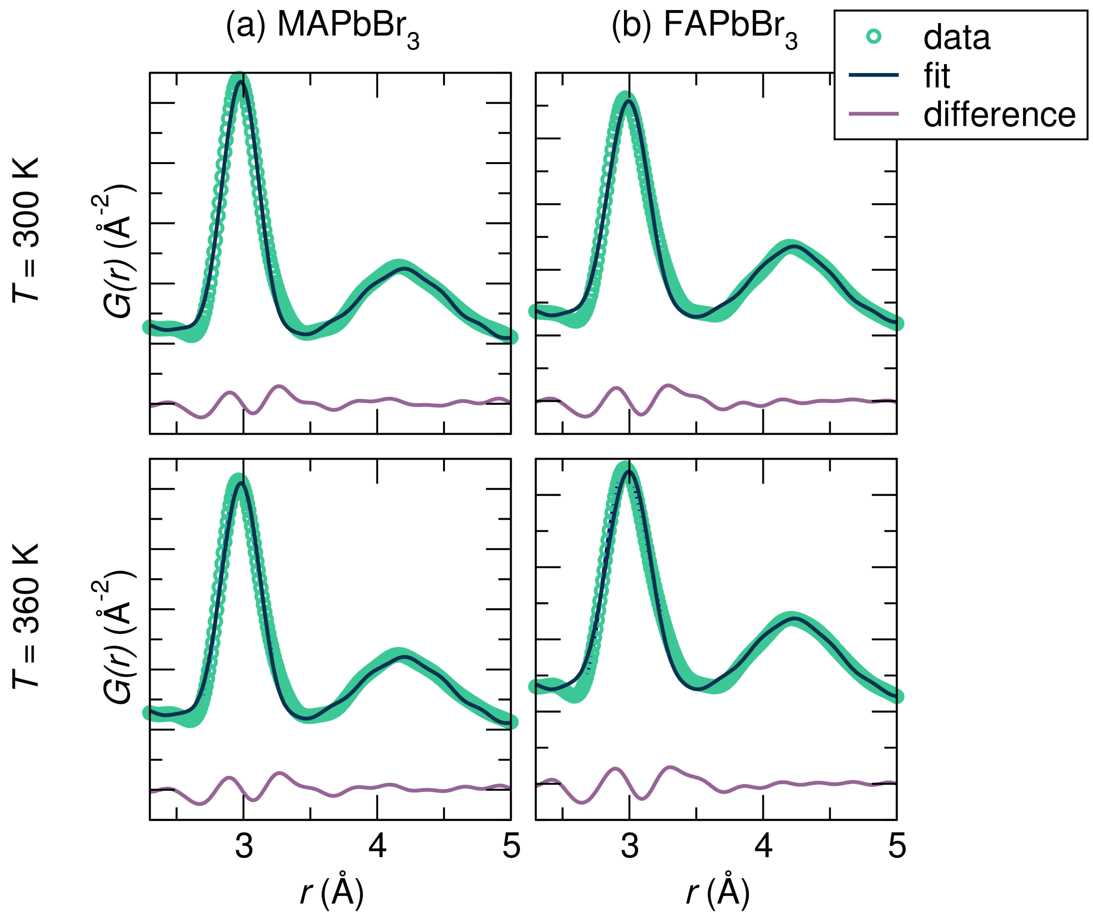

Figure 5: Fits of the synchrotron X-ray PDF data for (a)MAPbBr$_3$ and (b) FAPbBr$_3$ over an r-range of 2 Å to 5 Å against the cubic $Pm\bar{3}m$ models at 300 K (top row) and 360 K (bottom row). The difference curves for all fits indicate the cubic model does not accurately describe the shape of the first Pb–Br correlation at approximately 3 Å.

Rhombohedral fits of $A\mathrm{PbBr}_3$ at 300 K and 360 K

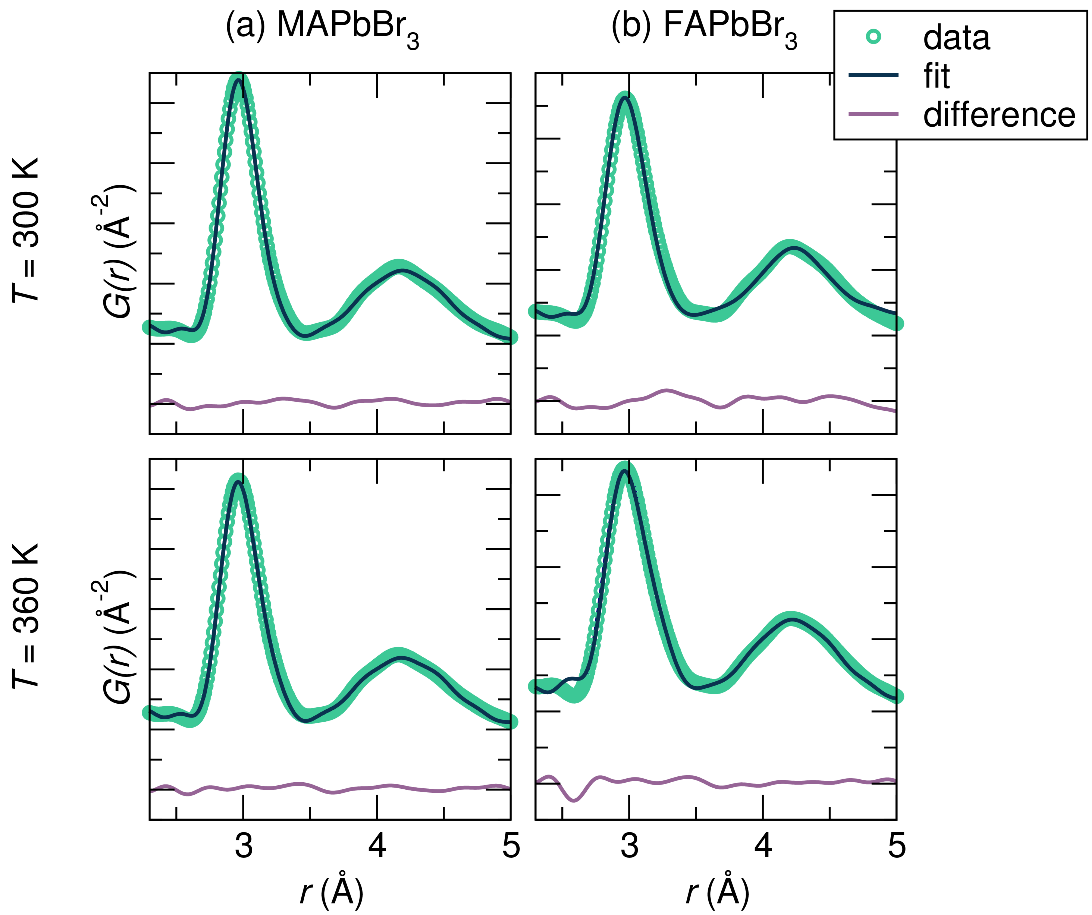

Figure 6: Fits of the synchrotron X-ray PDF data for (a)$\mathrm{MAPbBr_3}$ and (b) $\mathrm{FAPbBr_3}$ over an r-range of 2 Å to 5 Å against the rhombohedral $R3m$ models at 300 K (top row) and 360 K (bottom row). The difference curves for all fits indicate the rhombohedral model provides a good description of the shape of the first Pb–Br correlation at approximately 3 Å.
